# In Vitro Antifungal Drug Resistance Profiles of Clinically Relevant Members of the Mucorales (Mucoromycota) Especially with the Newer Triazoles

**DOI:** 10.3390/jof7040271

**Published:** 2021-04-02

**Authors:** Andrew M. Borman, Mark Fraser, Zoe Patterson, Michael D. Palmer, Elizabeth M. Johnson

**Affiliations:** 1 UK National Mycology Reference Laboratory, Public Health England, Science Quarter, Southmead Hospital, Bristol BS10 5NB, UK; Mark.Fraser@nbt.nhs.uk (M.F.); Zoe.Patterson@nbt.nhs.uk (Z.P.); Michael.Palmer@nbt.nhs.uk (M.D.P.); 2 Medical Research Council Centre for Medical Mycology (MRC CMM), University of Exeter, Exeter EX4 4QD, UK

**Keywords:** Mucoromycota, Mucorales, antifungal susceptibility testing, minimum inhibitory concentrations, amphotericin B, posaconazole, isavuconazole

## Abstract

Mucoromycoses (infections caused by members of the order Mucorales, phylum Mucoromycota [ex-Zygomycota]) are highly destructive, rapidly progressive infections, with dire prognoses especially when they occur in immunocompromised hosts. Current treatment guidelines recommend liposomal formulations of amphotericin B with adjunctive surgery as first line therapy, with the newer triazoles posaconazole or isavuconazole as alternative treatments, or as salvage therapy. Among the many organisms belonging to this order, a limited number of species in the genera *Rhizopus*, *Mucor*, *Lichtheimia* and *Rhizomucor* are responsible for most cases of human infection. Here, we present the minimum inhibitory concentration data (MICs) for amphotericin B, posaconazole, isavuconazole, itraconazole and voriconazole with a panel of over 300 isolates of the five most common agents of human infection (*Lichtheimia corymbifera*, *Rhizopus arrhizus*, *R. microsporus*, *Rhizomucor pusillus* and *Mucor* spp.) determined using the CLSI broth microdilution method. In agreement with previous studies, the most active antifungal drug for all Mucorales was amphotericin B, with MICs within the range that would predict susceptibility with *Aspergillus fumigatus*. Conversely, MICs for voriconazole against all species tested were high, and above the range associated with clinical efficacy with *A. fumigatus*. Interestingly, whilst isavuconazole and posaconazole MIC distributions indicated in vitro activity against some members of the Mucorales, activity was species-dependent for both agents. These data underscore the importance of accurate identification of the causative agents of mucoromycosis, coupled with antifungal susceptibility testing of individual isolates, in determining the optimal treatment of infections caused by these aggressive opportunistic human fungal pathogens.

## 1. Introduction

Mucoromycosis (ex-zygomycosis) is a relatively rare, but life-threatening infection that predominantly affects immunocompromised patients (patients with neutropenia, diabetic ketoacidosis or iron overload), or those suffering from severe burns or physical traumas [1,2,3,4], and is caused by members of the order Mucorales, phylum Mucoromycota [5]. These fungi, which were previously classified in the now-obsolete polyphyletic phylum Zygomycota, are characterized by their pauci-septate, broad, ribbon-like hyphae and extremely rapid growth in vitro and in vivo, with extensive angioinvasion, tissue necrosis and infarction and contiguous spread the characteristic presentations of infection [6]. Even with rapid diagnosis and appropriate medical management, infections are highly destructive and rapidly progressive and are associated with dire prognoses [6]. *Rhizopus*, *Mucor*, *Lichtheimia* and *Rhizomucor* are the genera most frequently associated with invasive human infections [1,2,3,4,6], with *Rhizopus* spp. accounting for approximately half of infections reported in Europe [2,7].

Successful treatment of all manifestations of mucoromycosis relies upon early complete surgical treatment and reversal of immune deficiencies or other pre-disposing factors (where clinically possible), together with systemic antifungal therapy using high doses of a liposomal formulation of amphotericin B, or alternatively isavuconazole or posaconazole where appropriate [6]. A variety of previous studies have demonstrated that amphotericin B exhibits good activity (relatively low minimum inhibitory concentrations [MICs]) against a variety of species in the Mucorales in vitro, supporting its recommendation as first line agent for treatment of infections with members of this order [8,9,10,11]. However, data on in vitro activity of posaconazole and especially isavuconazole against these filamentous fungi are more scant [10,11,12,13,14,15]. Moreover, the results of direct comparisons of the activity of these two triazoles in vitro are somewhat contradictory, with some studies showing roughly equivalent activities but species-specific variations [11,12], whilst others suggested that posaconazole MICs were generally lower than isavuconazole MICs with the common agents of mucoromycosis [13,14]. Additionally, it remains to be determined whether reported MIC differences between posaconazole and isavuconazole with members of the Mucorales are clinically relevant, given that drug exposures in vivo are usually higher with isavuconazole than with posaconazole [16,17,18,19].

Given the relatively diverse number of fungal pathogens within Mucorales, and reports of species-specific variations in antifungal susceptibility, in vitro antifungal susceptibility testing of individual isolates is recommended for epidemiological reasons, and albeit with more marginal support in order both to optimize treatment strategies and to detect resistant isolates. Moreover, despite the existence of standardized methodologies for the susceptibility testing of filamentous fungi [20,21], insufficient data exists to allow the proposal of species-specific interpretive breakpoints or epidemiological cutoff values (ECVs) for most of these important fungal pathogens [11,22]. Here, we present the in vitro susceptibility profiles for amphotericin B, itraconazole, voriconazole, posaconazole and isavuconazole with a panel of over 300 isolates representing the five most common species of Mucorales associated with human infections, with MICs determined using the CLSI broth microdilution methodology [20]. Such data are intended to contribute to the existing literature concerning in vitro potency of the three antifungal agents recommended as treatment options for mucoromycosis and also eventually to aid the future development of Epidemiological Cut-Off Values (ECVs) and Clinical Breakpoints (CBPs).

## 2. Materials and Methods

### 2.1. Clinical Isolates for MIC Distribution Analyses

MIC distributions were ascertained for a total of 365 clinical isolates of members of the Mucorales submitted to the UK National Mycology Reference Laboratory (MRL) for identification and susceptibility testing during the periods 2006–2016 (prior to inclusion of isavuconazole in antifungal susceptibility testing panels) and 2017–2020 (after implementation of isavuconazole MIC testing). Isolates included *Lichtheimia corymbifera* (*n* = 112, with 48 from the period 2017–2020), *Rhizopus arrhizus* (28, with 12 from the period 2017–2020), *R. microsporus* (96, with 46 from the period 2017–2020), *Rhizomucor pusillus* (37, with 5 from the period 2017–2020) and *Mucor* spp. (99, with 21 from the period 2017–2020). Isolates were identified phenotypically according to standard protocols in our laboratory, and identity of all isolates received during the period 2017–2020 was confirmed by MALDI-ToF MS analysis as described previously [23] using an extended in-house mass spectral profiles database created and curated by the MRL. The commercial Bruker Filamentous Fungus Database V3 contains mass spectral profiles for 8 genera of mucoraceous moulds, encompassing 19 different species in Mucorales. The in-house MRL database contains 10 genera, covering 15 species of Mucorales. Any isolates that could not be identified by MALDI-ToF or which did not exceed the criteria for robust identification (Mean LogScore > 2.000) were subjected to molecular identification. Since there was excellent concordance between phenotypic and proteomic identifications during this period (data not shown), we are confident in the reliability of the phenotypic identification methods that were employed in the period 2006–2016. MICs were determined according to CLSI guidelines [20] by broth microdilution as described below. For comparison, MIC data obtained with *Aspergillus fumigatus* and the same antifungal agents during the period 2019–2020 was also collated and included. Currently there is the largest MIC database and best correlation of MIC with outcome for this species, such that CBPs have been defined for some agents (EUCAST clinical breakpoint Table v10.0 [24]) that align closely with ECVs generated by CLSI methodology. Although breakpoints derived by one test method should not be applied to data derived by a different method, the MIC results generated by CLSI and EUCAST methods for mould isolates are generally much closer aligned than for yeasts for many of the antifungal agents included in the current study [25,26]. Suggested ECVs for *A. fumigatus* derived by CLSI testing align closely with the clinical breakpoints suggested by EUCAST for all of the agents tested in this study.

### 2.2. Antifungal Agents and Drug Concentration Ranges 

Antifungal drugs were obtained from their respective manufacturers as standard powders. Amphotericin B (Sigma Chemical Co., St. Louis, MO, USA), isavuconazole (Basilea Pharmaceutica Ltd., Basel, Switzerland) and voriconazole (Pfizer Central Research, Sandwich, UK) were dissolved in dimethyl sulfoxide. Itraconazole (Janssen Research Foundation, Beerse, Belgium) and posaconazole (Merck, Sharp and Dohme, Hoddesdon, UK) were dissolved in PEG400 by heating to 70 °C. Based on our own laboratory experience, the solubility of itraconazole and posaconazole is greater in PEG than in DMSO as recommended by CLSI, and precipitation of both agents upon freezing is reduced. Serial 2-fold dilutions of the various drugs were prepared in Roswell Park Memorial Institute (RPMI) 1640 medium (with L-glutamine, without bicarbonate; Sigma Chemical Co., St. Louis, MO, USA), and buffered to pH 7.0 using a 0.165 M solution of MOPS (Sigma Chemical Co., St. Louis, MO, USA).

### 2.3. CLSI Broth Microdilution Determination of Mould Minimum Inhibitory Concentrations (MICs)

MICs were determined in round-bottomed 96 well plates with mould conidial suspensions prepared in RPMI 1640 and adjusted to final concentration of 2.5 × 10^4^ CFU/mL as previously described [20]. All assays included *Aspergillus fumigatus* control isolates NCPF 7097 and NCPF 7100. Inoculated plates were incubated for 48 h at 35 °C. MICs were read at 24 (for *L. corymbifera*, *R. arrhizus*, *R. pusillus* and *R. microsporus*) or 48 h incubation according to current CLSI guidelines (i.e., the concentration of antifungal drug that elicited 100% inhibition of growth for all five study drugs).

### 2.4. Data Analysis

MIC ranges and the drug concentrations required to inhibit 50% (MIC_50_) or 90% of isolates (MIC_90_) were determined for all species that comprised at least 7 isolates. For species comprising less than 7 isolates, only MIC ranges were determined.

## 3. Results

The results of in vitro susceptibility testing of clinical isolates of Mucorales submitted to the MRL are shown as MIC distributions for amphotericin B, itraconazole, posacoanzole, voriconazole and isavuconazole in Table 1, Table 2, Table 3, Table 4 and Table 5, respectively, and MIC ranges, MIC50 and MIC90 values for the same organisms-antifungal agent combinations are summarized in Table 6. In all tests, the MICs of the control reference strains were within the limits accepted by CLSI (data not shown). Since CLSI wild-type MIC distributions and ECVs have yet to be proposed for many of the organism-antifungal agent combinations examined here, the MIC distributions obtained with clinical isolates of *Aspergillus fumigatus* over the same period were included for comparison. However, since a significant trend towards azole resistance was observed in isolates of *A. fumigatus* referred to the MRL over the study period, MIC values obtained with isolates of *A. fumigatus* for both the period 2019–2020 and also 2006–2016 are shown for comparison.

MIC data for amphotericin B (Table 1) were broadly in agreement with several previously published international studies [7,8,9,10,11,26,27] with the vast majority of isolates of all 5 species of Mucorales with MICs below the *A. fumigatus* ECV of 2 mg/L (367/370; 99.2%). Indeed, modal amphotericin B MICs reported here were identical (for *L. corymbifera*, *Mucor* spp., *R. arrhizus*, and *R. microsporus*) and within 1 doubling dilution (for *R. pusillus*) to those previously reported in a multicenter evaluation using CLSI methodologies that aimed to define ECVs for these organisms [22]. Moreover, in agreement with virtually all previous studies, the most active antifungal drug against all species of Mucorales was amphotericin B, with very little variation in MIC ranges, MIC_50_ or MIC_90_ values between different species or genera (Table 1 and Table 6), and only a single isolate of *Mucor* sp. with an MIC in excess of the amphotericin B ECVs proposed for these organisms (97.5% ECVs of 2, 2, 4 and 2 mg/L, for *L. corymbifera*, *M. circinelloides*, *R. arrhizus* and *R. microsporus*, respectively; [22]).

Although itraconazole is not recommended as a first line treatment for infections due to members of the Mucorales, it was included in the current analysis due to its structural and functional similarities with posaconazole [28]. Numerous recent studies have highlighted the emergence of azole resistance in isolates of *A. fumigatus*, which has been driven, at least in part, by environmental exposure to agricultural azole antifungal agents [29]. As mentioned above, we have also observed a significant trend towards azole resistance in isolates of *A. fumigatus* submitted to the MRL for susceptibility testing. Indeed, whilst less than 5% of isolates (109/2268) tested in the period 2006–2016 exhibited itraconazole MICs in excess of the ECV for A. fumigatus, 20% of isolates (27/135) referred in 2019–2020 had elevated itraconazole MICs (Table 2). On the basis of the same ECVs proposed for *A. fumigatus*, a significant proportion of the isolates of the various Mucorales species tested exhibited MIC values suggestive of some useful in vitro activity (Table 2 and Table 6). In agreement with previous studies [22,27], the itraconazole MICs for *Mucor* spp., *Rhizopus* spp. (and to a lesser extent *R. pusillus*) were significantly higher than those observed with L. corymbifera, with >95% of isolates of the latter species exhibiting MICs below the ECV for *A. fumigatus* with this antifungal agent, as compared to only approximately 40–50% of isolates of *Mucor* and *Rhizopus* spp. and 80% of isolates of *R. pusillus* (Table 2).

Posaconazole MIC distributions are presented in Table 3. Similar patterns were observed with posaconazole MIC ranges to those discussed above with itraconazole, with evidence of greater antifungal activity (as judged by the proportions of isolates with MICs below the ECV proposed for *A. fumigatus*) against isolates of *L. corymbifera* and *R. pusillus* than against *Mucor* and *Rhizopus* spp. However, it should be noted that modal MIC values with posaconazole were several doubling dilutions lower than the itraconzole equivalents for isolates of Rhizopus and Mucor spp. (compare Table 2 and Table 3; Table 6), suggesting that posaconazole does exhibit greater in vitro activity than itraconazole against all Mucorales. Once again, these MIC profiles are in good agreement with previous studies performed using CLSI methodologies [11,22,26,27], which reported species-specific differences in antifungal activity and significant inhibitory activity particularly against members of the Lichtheimiaceae (*L. corymbifera* and *R. pusillus*).

MIC distributions for voriconazole and isavuconazole are presented in Table 4 and Table 5, respectively. Although it is widely accepted that voriconazole has very limited activity against the Mucorales, it was included as a comparator in this study due to the structural similarities that its shares with isavuconazole [28]. In agreement with previous studies, voriconazole MICs with all members of the Mucorales tested far exceeded the proposed ECV for *A. fumigatus* and were significantly above the MIC range that correlates with clinical efficacy for *A. fumigatus* (Table 4), with little evidence of species-specific variation. For all species tested, voriconazole modal MICs, and MIC_50_ and MIC_90_ values were extremely elevated (Table 6), in keeping with the well-accepted lack of clinical efficacy with this agent. In contrast to voriconazole, isavuconazole exhibited detectable, but limited in vitro inhibitory activity against some members of the Mucorales. In excellent agreement with previous reports [13,14,30,31] approximately 25% of isolates *of L. corymbifera*, *R. arrhizus* and *R. microsporus* had MICs of less than 2 mg/L with isavuconazole (Table 5). However, for species of *Mucor* and *Rhizomucor*, MICs were significantly higher than the ECV proposed for *A. fumigatus* (1 mg/L) and generally exceeded the serum drug concentrations (2–4 mg/L) reported in most patients on isavuconazole therapy [18,19]. Indeed, MIC_50_ values with isavuconazole were 2 (*Rhizopus* spp.) and 4 doubling dilutions higher (*Mucor* spp., *L. corymbifera*) when compared to posaconazole, and MIC_90_ values followed a similar trend (Table 6).

## 4. Discussion

Ideally, antifungal susceptibility testing should provide a result that can be used to predict the likelihood of treatment success/failure of an infection by that organism with the antifungal agent under study. However, at best, MICs are a measure of in vitro potency of the particular antifungal agent, and prediction of treatment outcome requires much additional data, and importantly the generation of species-specific clinical breakpoints (CBPs) that allow categorization of MIC values. The development of CBPs for many of the rarer filamentous fungi (including the Mucorales) is currently impossible, due to a paucity of clinical data concerning treatment outcomes and the confounding issues of the impact of underlying conditions on the treatment outcomes. In the absence of CBPs, ECVs can be used to determine whether a given isolate has a “non-wild-type” response to antifungal agents that might be indicative of acquired resistance. Currently, ECVs have been proposed only for a limited number of Mucorales-antifungal agent combinations [22], due to insufficient in vitro MIC data. The current study, whilst not geared towards developing ECVs in its own right, will hopefully contribute to the growing literature and aid future development of ECVs and CBPs.

Since ECVS are only available for limited antifungal drug-Mucorales combinations (*M. circinelloides*, *L. corymbifera*, *R. arrhizus* and *R. microsporus* with amphotericin B and posaconazole, and *R. arrhizus* with itraconazole), here we have chosen to interpret MICs using the ECVs and clinical breakpoints proposed for *Aspergillus fumigatus*. This approach has been widely used previously (see for example [11]), and we believe that it is less confusing than interpreting a subset of MICs against organism-specific ECVs and the remainder against generic ones. In addition, here we have chosen to refer to the population of organisms with elevated MICs as “resistant” rather than as “non-WT” to avoid the additional confusion often encountered in microbiology laboratories where it is presumed that an organism with a “wild-type MIC” will automatically respond to that antifungal, despite the whole “WT population” being heavily skewed towards MIC values that are likely to reflect intrinsic resistance (see for example all of the MICs ranges for the Mucorales with voriconazole, Table 4).

The MIC distributions presented here for various antifungal drugs against members of the Mucorales are broadly concordant with those from a number of previous studies that suggested that while both posaconazole and isavuconazole possess in vitro anti-Mucorales activity, amphotericin B exerts the most potent antifungal activity against these organisms [11,12,13,14,15,22,26,30,31]. Additionally, the current data are in agreement with previous reports demonstrating that while amphotericin B possesses good activity against all Mucorales genera and species tested to date [10,11,26,32], the antifungal activities of itraconazole, isavuconazole and posaconazole are highly species-dependent, and voriconazole possesses no discernible in vitro activity against this group of organisms [11,12,13,14,15,26,32].

With the panel of Mucorales tested here, itraconazole, isavuconazole and posaconazole activity was highest against *L. corymbifera*, and lowest against *Mucor* spp., again in agreement with previous studies [11,12,13,14,15,22,26,27,32]. A recent study based on a revised species concept of *Mucor* species reported differences in antifungal susceptibility profiles for several species formerly grouped in *M. circinelloides*, in particular with the azole antifungal drugs [27]. Since a substantial proportion of the isolates of *Mucor* species included in the present study were identified prior to the recognition that *M. circinelloides sensu lato* contains several disparate species, we cannot refute or confirm this previous study based on the data presented here. However, proteomic identification of a subset of 31 isolates of *Mucor* species identified more recently in our laboratory demonstrated that *M. circenelloides sensu stricto* predominates in clinical specimens in the UK, with only 6 isolates identified as other *Mucor* species (*M. racemosus*, N = 2; *M. velutinosus*, N = 2; *M. plumbeus* and *M. indicus*, 1 isolate of each). No obvious differences in susceptibility profiles were evident across this albeit small selection of different *Mucor* species (data not shown). A potential limitation of the current study is that the identification of isolates analysed prior to 2017 was based upon phenotypic features rather than molecular or proteomic approaches, and thus might be prone to errors. However, as stated previously, isolates received during period 2017–2020 were identified by MALDI-ToF MS analysis and there was excellent concordance between phenotypic and proteomic identifications during this period (data not shown). Thus, we believe that we can be reasonably confident in the reliability of the phenotypic identification methods that were employed in the period 2006–2016. In addition, for *L. corymbifera* and *R. microsporus* a significant proportion of the included isolates (49/113 and 46/96, respectively) were received in the period 2017–2020, permitting a direct comparison of MIC distributions between organisms that had been identified phenotypically versus by MALDI ToF. Modal MICs and MIC distributions were very similar between the individual and combined datasets. Moreover, even with the smaller dataset that contained only isolates identified by MALDI-ToF, bimodal itraconazole MIC distributions were observed with *Mucor* spp. and *Rhizopus microsporus*, arguing against the idea that such bimodal distributions are due to the presence of multiple species that have been erroneously identified (data not shown).

Although the data concerning invasive mould infections is less compelling than the equivalent data for pathogenic yeasts, previous studies have proposed that outcomes of therapy may be improved when fungicidal as opposed to fungistatic agents are employed [33]. In the current study we have not directly evaluated whether the inhibitory effects of the tested antifungal agents against members of the Mucorales were fungicidal or fungistatic. However, previously published data suggest that while amphotericin B demonstrates some fungicidal activity against these fungi [10,32], the Minimum Fungicidal Concentrations observed with posaconazole and isavuconazole are generally extremely high [13,15,32], suggesting that these agents are likely to be fungistatic in vivo. Together with the observations here and elsewhere that amphotericin B demonstrates the most predictable in vitro activity against the Mucorales (in terms of low MICs that would be consistent with clinical success in infections with *A. fumigatus*), these data further support the historical designation of amphotericin B (and its lipid formulations) as first line agent of choice for the treatment of infections caused by members of the Mucorales [6,34,35].

While a limited number of individual studies have reported that isavuconazole and posaconazole exhibit roughly equivalent in vitro activities against the agents of mucoromycosis as evidenced by similar proportions of drug-organism MICs that exceeded the ECVs proposed for *A. fumigatus* [11,12], the majority of extant data suggests that posaconazole MICs with members of the Mucorales are generally lower than the equivalent isavuconazole MICs [13,14,26]. Our data are consistent with the consensus body of evidence, with the relative activities of the azole antifungals against the Mucorales being ordered posaconazole > isavuconazole > voriconazole according to the MIC distributions and ranges presented here and the proportions of drug-organism MICs exceeding the *A. fumigatus* ECVs proposed for each antifungal agent. Interestingly, based on absolute MIC values, MIC distributions and MIC_50_/MIC_90_ values in vitro, our data suggests that itraconazole is similar to posaconazole, and superior to isavuconazole with certain of these difficult to treat organisms, in particular *L. corymbifera* and *R. pusillus*. The in vitro activity of itraconazole against these organisms has been noted previously [reviewed in 35]. However, there is a lack of support for its usage in both historical and updated guidelines for the treatment of mucoromycosis [6,34,35], probably in part due to the issue of consistently achieving satisfactory therapeutic levels of this drug during treatment. However, direct comparisons of MIC values across different compounds are of limited value in determining potential clinical utility as the pharmacokinetic and pharmacodynamic parameters associated with clinical efficacy vary from drug to drug, as does bioavailability [19,31,36,37,38,39]. For instance, the AUC for isavuconazole is 3–6 times higher than that for posaconazole (depending on the formulation) and almost 10 times that for itraconazole. Given that the ratio of AUC:MIC is the best predictor of outcome for the azole antifungal agents, these differences in AUC are likely to at least partly offset the higher in vitro MICs observed with isavuconazole and explain the clinical efficacy of isavuconazole as compared to amphotericin B or posaconazole in the primary or salvage treatment of mucoromycosis [40,41,42,43] and the comparable activity of isavuconazole and posaconazole when used as treatment of mucoromycosis in an immunocompromised mouse model ([44] and references therein). Finally, a number of recent publications have reported breakthrough infections involving members of the Mucorales in haematology patients receiving isavuconazole or posaconazole prophylaxis [45,46,47,48,49,50], with similar species distributions reported with both antifungal agents again suggesting the possible equivalence of these two drugs.

## 5. Conclusions

In summary, here we have presented MIC distributions for amphotericin B, itraconazole, posaconazole, voriconazole and isavuconazole with in excess of 350 clinical isolates of filamentous moulds corresponding to 4 different genera of the Mucorales. For organism- antifungal drug combinations that had been studied previously elsewhere, the MIC ranges presented here are in excellent agreement with existing reports. The results of the current study underscore the excellent in vitro potency of amphotericin B against all members of the Mucorales, and the variable, species-specific activities of posaconazole and isavuconazole against these organisms. In addition, the present MIC data are intended to add to the existing body of literature with the hope of facilitating the development of ECVs for these organisms which would permit the detection of organism-drug combinations for which isolates display non-wildtype MICs.

## Figures and Tables

**Table 1:** Minimum inhibitory concentration (MIC) distributions for amphotericin B and key members of the Mucorales. The number of isolates is given in parentheses, together with the number of isolates with each given MIC value.* MIC values obtained with isolates of *Aspergillus fumigatus* for the period 2019–2020 are shown for comparison. ** %R denotes the proportion of isolates with non-wild-type MICs (i.e., MICs greater than the CLSI ECV or clinical breakpoint proposed for *A. fumigatus*, dashed line [24]). Modal MICs are indicated in bold, MIC_90_ values are underlined.

MIC (mg/L)											
Amphotericin B	0.03	0.06	0.125	0.25	0.5	1	2	4	8	≥16	%R **
*Aspergillus fumigatus* (201) *	–	–	22	**99**	63	17	–	–	–	–	0
*Lichtheimia corymbifera* (113)	1	5	13	42	**44**	8	–	–	–	–	0
*Mucor* sp. (99)	5	9	28	**29**	23	4	–	–	1	–	1.0
*Rhizopus* spp. (124)	–	3	18	41	**48**	14	1	–	–	–	0.8
*Rhizopus arrhizus* (28)	–	2	5	10	**10**	2	–	–	–	–	0.0
*Rhizopus microsporus* (96)	–	1	13	31	**38**	12	1	–	–	–	1.0
*Rhizomucor pusillus* (37)	1	1	7	**21**	7	–	–	–	–	–	0.0

**Table 2:** MIC distributions for itraconazole. For presentation conventions, see Legend to Table 1. * Since a significant trend towards azole resistance in isolates of *A. fumigatus* referred to the MRL was observed over the study period, MIC values obtained with isolates of *Aspergillus fumigatus* for both the period 2019–2020 and also 2006–2016 are shown for comparison %R values greater than 30% are indicated in bold face.

MIC (mg/L)												
Itraconazole		0.03	0.06	0.125	0.25	0.5	1	2	4	8	≥16	%R **
2006–2016 *	*Aspergillus fumigatus* (2268)	22	453	629	**632**	370	53	24	21	9	55	4.8
2019–2020 *	*Aspergillus fumigatus* (135)	–	4	27	**39**	34	4	7	8	7	5	20
	*Lichtheimia corymbifera* (84)	2	2	14	**26**	19	18	3	–	–	–	––
	*Mucor* sp. (88)	2	2	1	4	13	15	8	1	1	**41**	**58**
	*Rhizopus* sp. (88)	–	–	–	5	22	64	4	1	2	**28**	**39.8**
	*Rhizopus arrhizus* (25)	–	–	–	1	5	**6**	4	1	1	**7**	**52**
	*Rhizopus microsporus* (63)	–	–	–	4	17	20	0	–	1	**21**	**34.9**
	*Rhizomucor pusillus* (32)	2	4	–	**9**	8	3	3	–	–	3	18.8

**Table 3:** MIC distributions for posaconazole. For presentation conventions, see Legend to Table 2.

MIC (mg/L)												
Posaconazole		0.03	0.06	0.125	0.25	0.5	1	2	4	8	≥16	%R **
2006–2016 *	*Aspergillus fumigatus* (396)	111	**150**	65	37	21	8	2	–	1	1	8.3
2019–2020 *	*Aspergillus fumigatus* (187)	2	**65**	33	18	37	24	7	1	–	–	**36.9**
	*Lichtheimia corymbifera* (99)	-	3	18	**33**	32	12	1	-	-	-	**45.5**
	*Mucor* sp. (98)	3	3	4	9	18	**24**	13	8	2	14	**80.6**
	*Rhizopus* sp. (110)	-	1	3	18	**44**	21	9	3	1	10	**80**
	*Rhizopus arrhizus* (28)	-	-	2	4	**8**	**8**	2	-	-	4	**78.6**
	*Rhizopus microsporus* (83)	-	1	1	14	**36**	13	7	3	1	6	**80.5**
	*Rhizomucor pusillus* (35)	-	7	2	8	**12**	4	1	1	-	-	**51.4**

**Table 4:** MIC distributions for voriconazole. For presentation conventions, see Legend to Table 2.

MIC (mg/L)												
	**Voriconazole**	0.03	0.06	0.125	0.25	0.5	1	2	4	8	≥16	%R
2006–2016 *	*Aspergillus fumigatus* (2384)	1	13	309	**1631**	299	66	45	13	-	7	2.7
2019–2020 *	*Aspergillus fumigatus* (209)	1	2	4	31	**96**	20	29	17	4	5	26.3
	*Lichtheimia corymbifera* (84)	-	-	-	-	-	-	7	12	17	**48**	**100**
	*Mucor* sp. (86)	-	-	2	-	-	-	1	3	5	**75**	**97.7**
	*Rhizopus* sp. (83)	-	-	-	-	-	-	3	18	**32**	30	**100**
	*Rhizopus arrhizus* (23)	-	-	-	-	-	-	2	3	8	**10**	**100**
	*Rhizopus microsporus* (60)	-	-	-	-	-	-	1	15	24	**20**	**100**
	*Rhizomucor pusillus* (32)	-	-	1	-	-	3	1	3	5	19	**87.5**

**Table 5:** MIC distributions for isavuconazole. For presentation conventions, see Legend to Table 2. Since isavuconazole testing was not employed prior to 2017, historical data for *A. fumigatus* isolates for the period 2006–2016 is not available for comparison.

MIC (mg/L)												
	**Isavuconazole**	**≤0.03**	0.06	0.125	0.25	0.5	1	2	4	8	≥16	%R *
2019–2020 *	*Aspergillus fumigatus* (339)	-	2	2	70	**92**	76	41	33	11	12	28.6
	*Lichtheimia corymbifera* (28)	-	-	1	-	2	3	4	8	3	7	**78.6**
	*Mucor* sp. (21)	-	-	-	-	-	-	-	4	2	15	100
	*Rhizopus* sp. (35)	-	-	-	-	-	9	**10**	8	3	5	**74.3**
	*Rhizopus arrhizus* (12)	-	-	-	-	-	**3**	**3**	1	1	**4**	**75**
	*Rhizopus microsporus* (23)	-	-	-	-	-	6	**7**	**7**	2	1	**73.9**
	*Rhizomucor pusillus* (5)	-	-	-	-	-	-	-	**4**	1	-	**100**

**Table 6:** Summary of MIC data for isolates of the Mucorales. NA—not appropriate due to the small number of isolates tested for this species.

	MIC (mg/mL)			
Species	Antifungal Agent	Range	MIC_50_	MIC_90_
*L. corymbifera*	Amphotericin B	0.03–1	0.25	0.5
	Itraconazole	0.03–2	0.25	1
	Posaconazole	0.06–2	0.25	1
	Voriconazole	2–>16	>16	>16
	Isavuconazole	0.125–>16	4	>16
*Mucor* sp.	Amphotericin B	0.03–8	0.25	0.5
	Itraconazole	0.03–>16	2	>16
	Posaconazole	0.03–>16	1	>16
	Voriconazole	0.125–>16	>16	>16
	Isavuconazole	4–>16	>16	>16
*Rhizopus arrhizus*	Amphotericin B	0.06–2	0.25	0.5
	Itraconazole	0.25–>16	2	>16
	Posaconazole	0.125–>16	0.5	8
	Voriconazole	2–>16	8	>16
	Isavuconazole	1–>16	2	>16
*R. microsporus*	Amphotericin B	0.06–2	0.5	1
	Itraconazole	0.25–>16	1	>16
	Posaconazole	0.06–>16	0.5	4
	Voriconazole	2–>16	8	>16
	Isavuconazole	1–>16	2	8
*Rhizomucor pusillus*	Amphotericin B	0.03–0.5	0.25	0.5
	Itraconazole	0.03–>16	0.5	2
	Posaconazole	0.06–4	0.5	1
	Voriconazole	0.125–>16	>16	>16
	Isavuconazole	4–8	NA	NA

## Data Availability

Data is contained within the article.
